# *Latilactobacillus sakei* Furu2019 and stachyose as probiotics, prebiotics, and synbiotics alleviate constipation in mice

**DOI:** 10.3389/fnut.2022.1039403

**Published:** 2023-01-05

**Authors:** Yanan Guo, Liqiong Song, Yuanming Huang, Xianping Li, Yuchun Xiao, Zhihuan Wang, Zhihong Ren

**Affiliations:** State Key Laboratory for Infectious Disease Prevention and Control, Research Units of Discovery of Unknown Bacteria and Function (2018 RU010), Chinese Center for Disease Control and Prevention, National Institute for Communicable Disease Control and Prevention, Chinese Academy of Medical Sciences, Beijing, China

**Keywords:** slow transit constipation, *Latilactobacillus sakei*, stachyose, synbiotics, intestinal flora

## Abstract

**Introduction:**

Slow transit constipation (STC) is a common disorder in the digestive system. This study aimed to evaluate the effects of stachyose (ST) and *Latilactobacillus sakei* Furu 2019 (*L. sakei*) alone or combined on diphenoxylate-induced constipation and explore the underlying mechanisms using a mouse model.

**Methods:**

ICR mice were randomly divided into five groups. The normal and constipation model groups were intragastrically administrated with PBS. The ST, *L. sakei*, and synbiotic groups were intragastrically administrated with ST (1.5 g/kg body weight), alive *L. sakei* (3 × 10^9^ CFU/mouse), or ST + *L. sakei* (1.5 g/kg plus 3 × 10^9^ CFU/mouse), respectively. After 21 days of intervention, all mice except the normal mice were intragastrically administrated with diphenoxylate (10 mg/kg body weight). Defecation indexes, constipation-related intestinal factors, serum neurotransmitters, hormone levels, short-chain fatty acids (SCFAs), and intestinal microbiota were measured.

**Results:**

Our results showed that three interventions with ST, *L. sakei*, and synbiotic combination (ST + *L*. sakei) all alleviated constipation, and synbiotic intervention was superior to ST or *L. sakei* alone in some defecation indicators. The RT-PCR and immunohistochemical experiment showed that all three interventions relieved constipation by affecting aquaporins (AQP4 and AQP8), interstitial cells of Cajal (SCF and c-Kit), glial cell-derived neurotrophic factor (GDNF), and Nitric Oxide Synthase (NOS). The three interventions exhibited a different ability to increase the serum excitatory neurotransmitters and hormones (5-hydroxytryptamine, substance P, motilin), and reduce the serum inhibitory neurotransmitters (vasoactive intestinal peptide, endothelin). The result of 16S rDNA sequencing of feces showed that synbiotic intervention significantly increased the relative abundance of beneficial bacteria such as *Akkermansia*, and regulated the gut microbes of STC mice. In conclusion, oral administration of ST or *L. sakei* alone or combined are all effective to relieve constipation and the symbiotic use may have a promising preventive effect on STC.

## Introduction

Slow transit constipation (STC) is a common clinical symptom of gastrointestinal dysfunction and is characterized by persistent dry stools, difficult bowel movements, and infrequent or incomplete defecation (1, 2). Constipation commonly occurs in the elderly, women, and people with high stress (3, 4). The overall incidence of constipation in the world is about 14%, and the incidence of constipation varies in different regions (5). In some parts of Africa, the incidence of constipation is as high as 30% (5). Mild constipation may induce hemorrhoids and anal fissures, and severe constipation may induce proctitis and even colorectal cancer (6). In people with heart disease, high blood pressure, and cirrhosis, constipation can even cause cardiac arrest, ruptured blood vessels, and massive bleeding (7). In addition, long-term constipation may lead to mental and psychological problems and severely affect people's health and quality of life (8).

Gut motility is controlled by several factors. The enteric nervous system (ENS) is the primary regulator of gut motility, followed by the autonomic nervous system (ANS) and central nervous system (CNS) (9). Both the ENS and CNS can produce 5-hydroxytryptamine (5-HT), which is a key neurotransmitter and mediates enteric nervous reflexes to initiate secretion and propulsive motility and acts on vagal afferents to regulate gut mortality (10). The ENS can interact with the gut microbiota *via* serotonin 5-HT (11). Gut microbiota and the metabolic products of bacterial fermentation are critical to the maturation and stimulation of the ENS then affect gut transit (12). Although it has been reported that constipation can change the composition of gut microbiota (13), several studies have shown that constipation could be relieved by regulating the intestinal microbiota and the metabolites (14), promoting gut-brain communication (15), and enhancing the intestinal peristalsis *via* the c-Kit pathway of interstitial cells of Cajal (ICCs) (9). ICCs are regarded as the pacemaker of gastrointestinal peristalsis and constipation is often accompanied by abnormalities in ICCs (16).

The prebiotics that has been confirmed so far mainly include oligosaccharides, dietary fiber, inulin, and others (17), which can promote specific beneficial bacteria growing rapidly in the gut. Stachyose (ST) belongs to the raffinose family of oligosaccharides and is formed by combining two α-galactoses with 1,6-glycosidic bonds on the glucosyl side of sucrose (18). Studies have shown that ST can relieve the symptoms of constipation in patients and significantly enhance the amount of Bifidobacteria and Lactobacilli and reduce the fecal *Clostridium perfringens* concentration (19). ST has been clinically proven to relieve constipation and it acts mainly by increasing the abundance of short-chain fatty acids (SCFAs) (20). *Latilactobacillus sakei*, Gram-positive and facultative anaerobic bacteria, was first discovered in Japanese sake (21). *L. sakei* is often used in fermented foods and has also been isolated from homemade fermented bean curd, kimchi, pig ears, and other local specialties in Sichuan (22). *L. sakei* Furu 2019 (*L. sakei*) was isolated from a traditional Chinese food called fermented bean curd which was considered to be beneficial “Chinese cheese.” Therefore, *L. sakei* is usually regarded as a transient resident of the human gastrointestinal tract because it can survive under harsh conditions. *L. sakei* has been reported to have several health benefits. *L. sakei* is currently known to have anti-inflammation and weight loss effects, for example, *L. sakei* ADM14 can alleviate high-fat diet-induced obesity and alter the gut microbiota in mice (23). *L. sakei* WIKIM31 can prevent the weight gain induced by the high-fat diet by modulating lipid metabolism and suppressing inflammation (24). *L. sakei* S1 can improve trinitrobenzene sulfonic acid-induced colitis by inhibiting NF-kappaB signaling in mice (25, 26).

Clinically, constipation is often treated with laxatives such as cisapride. However, drug treatment often has side effects (27). Thus, it is of need to develop an effective and safe treatment to relieve constipation. Some probiotic products have been used to treat constipation, such as live *Bacillus coagulans* capsules and *Bifidobacteria* capsules (28). The important advantage of these probiotics is that probiotics are gentler and safer (28). The effects of probiotics on the composition of the microbiota, SCFA production, and gut motility of constipation are still relatively poorly understood. Some prebiotics such as oligosaccharides could also alleviate constipation (29). It has been proposed that synbiotics may exert a better effect on host health (30). Since there is no information available thus far regarding the effect of *L. sakei* or ST alone or combined on constipation, we conducted this study to evaluate the efficacy of *L. sakei* and ST as probiotics, prebiotics, and synbiotics in alleviating constipation in a mouse model and also to further investigate the underlying mechanisms.

## Materials and methods

### Ethics statement

This study was approved by the Ethics Review Committee of the National Institute for Communicable Disease Control and Prevention at the Chinese Center for Disease Control and Prevention (Beijing, China).

### Reagents and preparation of *L. sakei*

Diphenoxylate-atropine containing 2.5 mg of diphenoxylate hydrochloride and 0.025 mg of atropine sulfate per tablet (H22022037, Changhong Pharmaceutical Co., Ltd, Changchun, China) was used to establish the constipation mouse model in this study. The ingredients of Stachyose (ST) used in this study include stachyose (no <70%), sucrose (12 ± 2%), raffinose (5 ± 1%), mannotriose (2 ± 0.5%), fructose (2 ± 0.5%), glucose (1 ± 0.2%), verbascose (2 ± 0.5%), water (no more than 5%), and 1% ash. ST was provided by the China National Research Institute of Food and Fermentation Industries. A stock solution (0.5 g/kg body weight) of ST in sterile PBS was microfiltered (0.22 μm sterile disc, Merck Millipore Ltd, USA) and kept at 4°C before use. Ten grams of gum Arabic (Beijing Lebesi des Biotechnology Co., Ltd., ALBJ1L) was added into 80 mL of ultrapure water. Then 10 g of activated carbon powder (Beijing Pringle Science and Trade Co., Ltd., C7261) was added into the Arabic solution and boiled three times to make the final 100 mL ink stock solution as an indicator of defecating the first black stool. *L. sakei* was isolated from a traditional Chinese food called fermented bean curd and was routinely grown in Man-Rogosa-Sharpe (MRS) medium in a 37°C carbon dioxide incubator (Forma CO2, Thermo Fisher Scientific, Waltham, MA, USA). A culture of *L. sakei* in the logarithmic growth phase was washed and resuspended with sterile PBS for oral inoculation of mice.

### Animal experiment

SPF male ICR mice (24–26 g, 6-week-old) were purchased from Beijing Vital River Laboratory Animal Technology Co., Ltd. (Beijing, China). Mice were housed in the Animal Center of China CDC and had free access to water and food under a 12/12-h dark/light cycle at a constant temperature of 22–24°C. After 7 days of acclimation, 40 mice were randomly divided into five groups: normal, constipation, prebiotics, probiotics, and synbiotics. The normal and constipation model groups were given 0.3 mL of PBS every morning. The prebiotics, probiotics, and synbiotics groups were intragastrically administrated 0.3 mL of ST (1.5 g/kg), alive *L. sakei* (3 × 10^9^ CFU/mouse), and the mixture of ST and *L. sakei* (1.5 g/kg plus3 × 10^9^ CFU/mouse), respectively. After 21 days of intervention, all mice were fast for 16 h. On day 22, all mice except the normal mice were intragastrically administrated with diphenoxylate (10 mg/kg) to establish the constipation mouse model according to previous publications (31, 32). After 30 min, all mice were gavaged with 0.25 mL of ink. Then mice were given the water and diet and put in metabolic cages individually. Within 5 h after oral administration of the ink, defecation index was recorded. After 16 h-fast, on day 23, fresh feces were collected for 16S rRNA sequencing. Then all mice except the normal mice were given 0.3 mL of diphenoxylate (5 mg/kg). After 25 min, all mice were sacrificed and the entire bowel was removed and its length was measured. The blood was collected to obtain serum for quantification of neurotransmitters. The colons were collected and kept in 4% paraformaldehyde and RNAlater (Thermo Fisher Scientific, Waltham, MA, USA), respectively. Fresh cecal content was collected and preserved at −80°C for quantification of SCFA_S_.

### Determination of defecation index in mice

On day 22, within 5 h after oral administration of the ink, the defecation index including the time of expelling black stool for the first time, the number of excreted feces, and the wet weight of the feces were recorded. Collected feces were continuously dried in an oven at 100°C for 2 h, and the dry weight of feces was measured to calculate the moisture content of the feces using the following formula (33).


(1) $$\begin{array}{l} R=\frac{W_{\left(wet\right)}-W_{\left(dry\right)}}{W_{\left(wet\right)}}\times 100\% \end{array}$$

### Determination of intestinal propulsion index in mice

The entire bowel was removed and its length was measured. The following formula was used to calculate the intestinal propulsion rate where S_1_ represents the total length of the intestinal tract, and S_2_ represents the distance from the pylorus to the front end of the ink.


(2) $$\begin{array}{l} D=\frac{S_{2}}{S_{1}}\times 100\% \end{array}$$

### Quantitative PCR detection of mRNA expression

Total RNA of the colonic tissues was extracted using TRIzol (Invitrogen, US) and the reverse transcription reactions were performed to generate cDNA by reverse transcription kit (Takara, Japan) according to the manufacturer's instructions. The cDNA products were subjected to RT q-PCR in an ABI 7500 Real-Time PCR System (ABI, United States). The amplification reaction conditions were as follows: pre-denaturation at 95°C for 30 s, denaturation at 95°C for 5 s, and annealing at 60°C for 30 s followed by 40 cycles. The relative levels of mRNA expression were calculated using the delta-delta CT method. Duplicated wells were set up for each sample and the average Ct value was determined from two runs. The gene expression level of β-actin was used as the internal control. ΔCt = Ct (target gene) – Ct (control gene), and relative gene expression was calculated as ΔΔCt = ΔCt (treated)–ΔCt (blank). The relative gene expression levels were converted to 2^−ΔΔCt^. The primers are listed in Table 1.

**Table 1:** List of primers used in the qRT-PCR assay for constipation-related intestinal factors.

**Gene**	**Primer sequence**
*β-Actin*	Forward: 5′-CGGACACGGACAGGATTGACA
	Reverse: 5′-CCAGACAAATCGCTCCACCAACT
*Aqp4*	Forward: 5′-GCAGACAAGGTGCAACGTGGTT
	Reverse: 5′-GGCGGAAGGCAAAGCAGTATGG
*Aqp8*	Forward: 5′-AGCAGGAGCAGGTGGCAGAA
	Reverse: 5′-TCCAAAGGCACGAGCAGGGT
*GDNF*	Forward: 5′-GGGGTATGGAGAAGTTGGCTAG
	Reverse: 5′-CTATGAGAATGCTGCCGAAAA
*SCF*	Forward: 5′-TCAGGGACTACGCTGCGAAAG
	Reverse: 5′-AAGAGCTGGCAGACCGACTCA
*c-Kit*	Forward: 5′-CATAGCCCAGGTAAAGCACAAT
	Reverse: 5′-GAACACTCCAGAATCGTCAACTC
*NOS*	Forward: 5′-TCAGCGGTGA TAGGA TAAAGCA
	Reverse: 5′-CGCTGTGCTAAGTAGCCCTCG

### Immunohistochemical analysis

The colons were fixed with 4% formaldehyde. Sections were first deparaffinized in xylene and passed through various concentrations of alcohol. Sections were incubated with 5% BSA (Wuhan Boster, China) for 30 min. The primary antibodies including anti-c-Kit antibody (CST, USA), anti-SCF antibody (Absin, China), anti-AQP4 antibody (Abcam, USA), and anti-AQP8 antibody (Absin, China) were added and incubated overnight at 4°C. Then the sections were rewarmed at room temperature for 1 h and washed three times with PBS buffer. The secondary antibody horseradish peroxidase-conjugated goat anti-rabbit IgG (Beyotime, China) was added dropwise and incubated at 37°C for 30 min. After washing 3 times with PBS for 10 min, DAB color development was performed. Hematoxylin was then used for counterstaining, differentiation, and reverse blue. After dehydration, washing, and mounting, the sections were photographed with Computer image processing system CMOS (OLYMPUS,Japan)and analyzed using Image-Pro Plus software (Media Cybernetic, US). The intensity of positive immunostaining and tissue area were determined by human-computer interaction. The average optical density was calculated as the ratio of integrated optical density versus tissue area. These results were evaluated by an experienced person who was blind to the samples in terms of treatment. The average optical density was statistically analyzed using GraphPad Prism5.

### Quantification of SCFAs

SCFA_S_ were determined by Beijing Nuohezhiyuan Biotechnology Co., Ltd. Briefly, 30 mg of mouse cecum content was suspended in 900 μL of 0.5% phosphoric acid (Sinopharm Chemical Reagent Co. Ltd., Shanghai, China). After centrifugation at 14,000 g 4°C for 10 min, 800 μL supernatant was mixed with an equal volume of ethyl acetate. After the mixture was centrifuged at 14,000 g for 10 min, 600 μL of the upper organic phase was collected for GC-MS analysis. Six hundred microliters of a mixed standard solution composed of acetic acid, propionic acid, butyric acid, isobutyric acid, valeric acid, isovaleric acid, and hexanoic acid with eight concentrations were added with 25 μ L 500 μM Methyl valerate as internal standard. The injection volume was 1 μL, and the split ratio was 10:1. Temperature program was as follows: the initial temperature was set at 90°C; the temperature was increased to 120°C at 10°C/min; then the temperature was increased to 150°C at 5°C/min; finally, the temperature was increased to 250°C at 25°C/min and maintained for 2 min. The carrier gas was helium and the carrier gas flow rate was 1.0 mL/min. Agilent 7890A/5975C GC-MS (Thermo Fisher Scientific Inc., Waltham, MA, USA) equipped with an Agilent DB-WAX (Agilent Technologies, Co., Ltd., US) capillary column (30 m × 0.25 mm ID × 0.25 μm) was used to determine the SCFA contents. MSD ChemStation software was used to obtain the chromatographic peak area and retention time. The contents of short-chain fatty acids in the samples were calculated based on the standard curve.

### Quantification of neurotransmitters

Serum was collected to measure 5-hydroxytryptamine (5-HT), vasoactive intestinal peptide (VIP), substance P (SP), motilin (MTL), and endothelin (ET) using serum test kits (Beijing Yisheng Zhaobo Biotechnology Co., Ltd.). Briefly, a total of 50 μL standard substance or 1:5 diluted samples were added on the enzyme-labeled coating plate, respectively, and the plates were sealed and incubated at 37°C for 30 min. Then the plates were washed 6 times with a washing solution. Fifty microliters of enzyme-labeled reagent was added into each well except for control wells. After incubation and washing, 50 μL of chromogenic reagents A and B were added and mixed then placed in the dark at 37°C for 10 min to develop color. Finally, 50 μL of stop solution was added into each well then the absorbance (OD value) was measured at 450 nm wavelength.

### 16S rRNA sequencing and intestinal microbiota analysis

Fresh fecal samples were collected as previously described and 16S rRNA sequencing was performed on the V3–V4 gene region to analyze the mouse fecal microbiota. MagPure Stool DNA KF kit B (Magen, China) was used to extract the DNA from feces. DNA was quantified using the Qubit dsDNA BR Assay Kit (Invitrogen, USA) and the quality was checked by agarose gel electrophoresis. The variable region V3–V4 of the bacterial 16S rRNA gene was amplified with the degenerate PCR primers 341F (5′-ACTCCTACGGGAGGCAGCAG-3′) and 806R (5′-GGACTACHVGGGTWTCTAAT-3′). Libraries were characterized by an Agilent 2100 Bioanalyzer (Agilent, USA). The amplicons and quality control of the raw data were conducted on the Illumina Hiseq 2500 platform (BGI, China). Clean data was performed using USEARCH (V10.0.240)5 and VSEARCH (V2.8.1)6 for reads splicing, filtering of low-quality reads, chimera removal, and operable taxonomic unit (OTU) construction. The α diversity and principal coordinates analysis (PCoA) was calculated and visualized by R software (V3.6.1; https://github.com/microbiota). Differential OTUs were identified and statistically analyzed by LEfSe (http://huttenhower.sph.harvard.edu/galaxy/) online using non-parametric factorial Kruskal–Wallis and Wilcoxon rank-sum tests. The threshold was set at 2.0 for discriminative features. The original data can be downloaded from the NCBI SRA database (PRJNA877764).

### Statistical analysis

Statistical analysis of data was performed with GraphPad Prism 5 software (GraphPad Inc., San Diego, CA, USA). All continuous variables were presented as mean ± standard deviation (SD), and category variables were expressed as *n* (%). Statistical differences between groups were determined by one-way analysis of variance (ANOVA) for normal distribution data. Otherwise, the non-parametric Kruskal–Wallis rank-sum test was used followed by Duncan's test for multiple comparisons for alpha diversity analysis of the gut microbiota. *P* < 0.05 was considered statistically significant.

## Result

### Effects of ST or *L. Sakei* as prebiotics, probiotics, and synbiotics on defecation parameters and gastrointestinal transit

**Figure 1:**
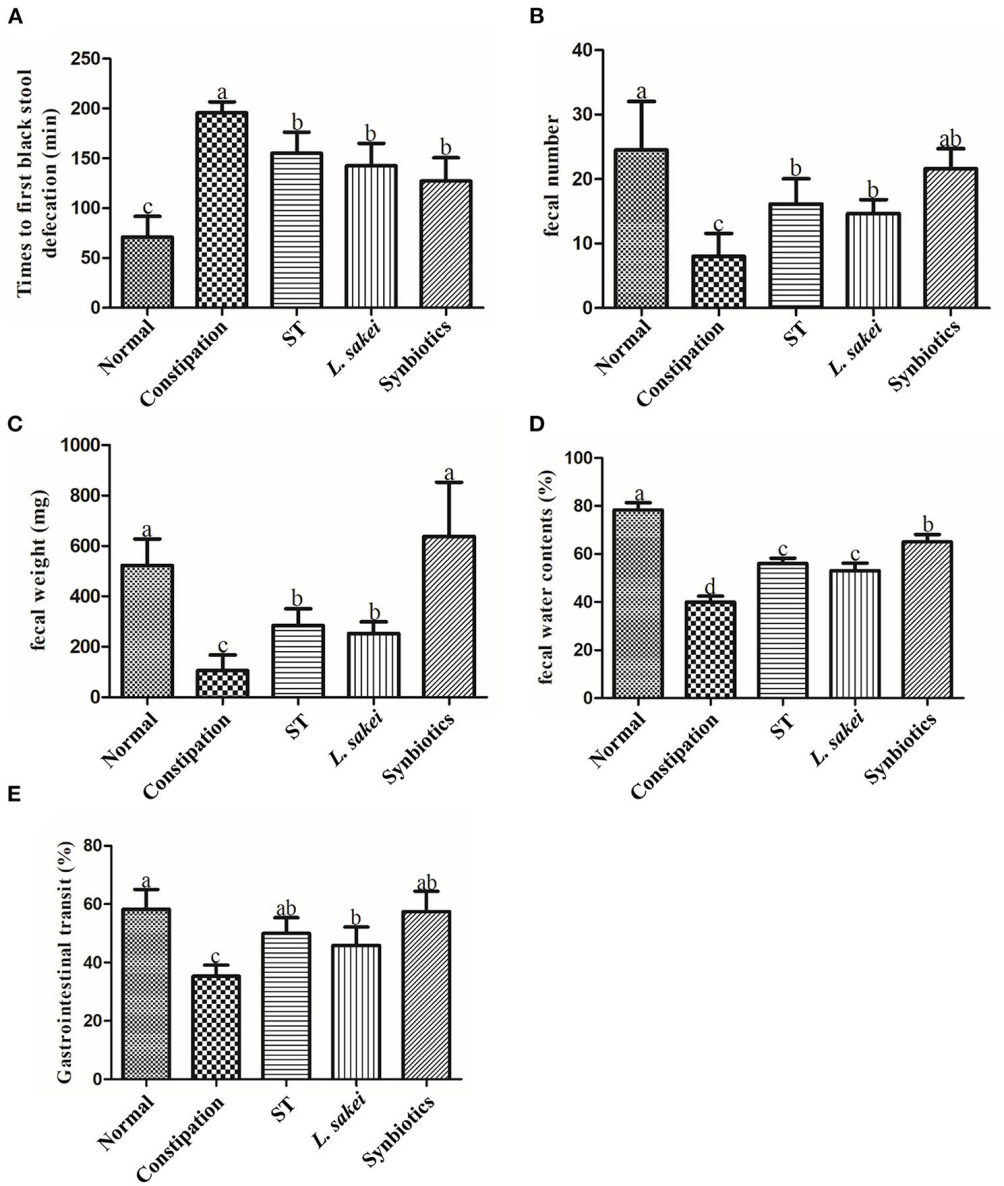
Effects of ST or *L. sakei* alone or combined on defecation parameters and gastrointestinal transit in mice. **(A)** Times to first black stool defecation; **(B)** Fecal number; **(C)** Fecal weight; **(D)** Fecal water contents; **(E)** Gastrointestinal transit. Data represent the mean ± SD (*n* = 6). Statistical analysis was conducted using One-way ANOVA followed by Tukey's multiple comparisons test for each group. The bars bearing different letters indicate a significant difference, *P* < 0.05.

The constipation-related indices in the diphenoxylate-induced constipation mouse model include the fecal weight, fetal number, fecal water content, the first black fecal defecation time, and gastrointestinal transit. The shorter time of defecating the first black stool indicates more rapid motility throughout the intestine and a stronger intestinal transport capacity. The higher GI transit rate indicates greater small intestinal motility. In this study, diphenoxylate treatment effectively induced constipation model as indicated by lower feces weight (105 ± 62.7 vs. 468.67 ± 162.96 mg), fewer feces number (8 ± 3.58 vs. 24.5 ± 7.56), less water content of feces (40 ± 6.1 vs. 78.33 ± 7.47%), longer time of first black feces defecation (70.83 ± 20.84 vs. 195.83±10.96 min), and lower gastrointestinal transit rate (35.33 ± 3.77 vs. 58.23 ± 6.79%) compared with the normal control mice (Figures 1A–E). After intervention with ST or *L. sakei* alone or combined, the five constipation-related indices in the three intervention groups were all significantly different from the constipation model group and close to the normal control group (Figures 1A–E). For example, the feces weight in the ST group (284.83 ± 66.62 mg), *L. sakei* group (252.8 ± 45.98 mg), and synbiotic group (552.4 ± 267.16 mg) were significantly higher than that in the constipation model group (105 ± 62.7 mg; *P* < 0.05). These results indicate that while all three interventions can alleviate the symptoms of constipation, the synbiotic combination of ST and *L. sakei* is more effective in some indicators than ST or *L. sakei* intervention.

### Effects of ST or *L. sakei* alone or combined on mRNA expression levels of constipation-related intestinal factors

**Figure 2:**
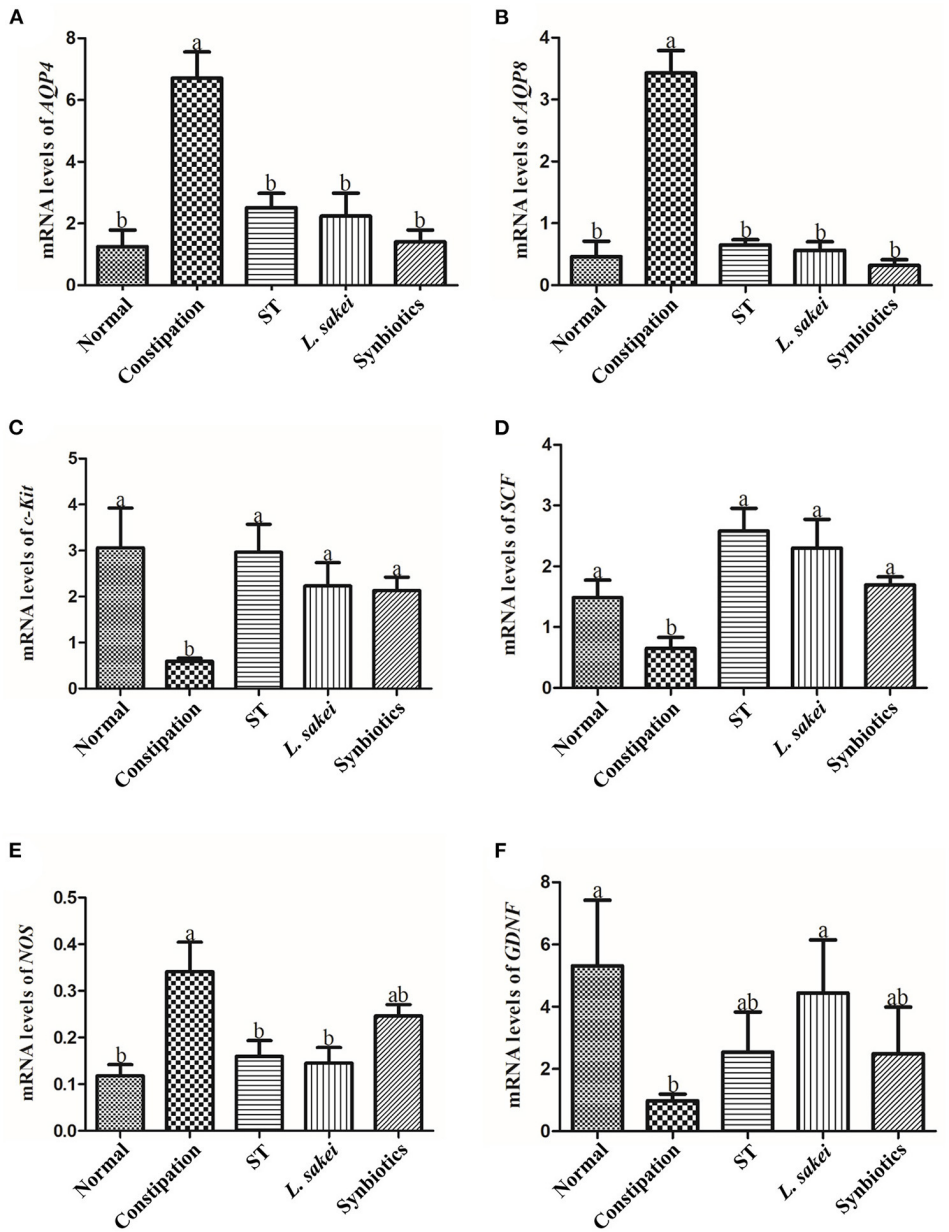
The effect of ST or *L. sakei* alone or combined on the mRNA expression levels of constipation-related intestinal factors in mice. **(A)** Changes in mRNA levels of *AQP4*; **(B)** Changes in mRNA levels of *AQP8*; **(C)** Changes in mRNA levels of *c-Kit*; **(D)** Changes in mRNA levels of *SCF*; **(E)** Changes in mRNA levels of *NOS*; **(F)** Changes in mRNA levels of *GDNF*. Data represent the mean ± SD (*n* = 6). Statistical analysis was conducted using One-way ANOVA followed by Tukey's multiple comparisons test for each group. Bars bearing different letters indicate a significant difference, *P* < 0.05.

Aquaporin (AQP) channels play a central role in regulating fluid homeostasis in the colon. Several AQP channels were detected in human colon epithelial cells. AQPs are primarily expressed in human colon epithelial cells. Our results showed that *AQP4* and *AQP8* expression in the colon of constipation mice was significantly higher than that of normal mice, while *AQP4* and *AQP8* expression was significantly down-regulated in the colon of three intervention groups (Figures 2A,B). In addition, activation of the C-kit receptor on the surface of interstitial Cajal cells (ICC) is closely related to ICC function, and the stem cell factor (SCF)/C-kit signaling pathway plays an important role in stimulating intestinal motility. We found that *c-Kit* and *SCF* expression in the colon of constipated mice was significantly lower than that of normal mice. The levels of *SCF* and *c-Kit* in ST or *L. sakei*, and synbiotics groups were all significantly higher than those in the constipation group (*P* < 0.05). The differences among the three intervention groups were not significant (Figures 2C,D, *P* > 0.05). The increasing expression of *GDNF* (glial cell-derived neurotrophic factor) in mice helps gastrointestinal innervation, which affects intestinal motility and avoids constipation. Our results showed that the expression of *GDNF* in the constipation group was significantly lower than that in the normal group (*P* < 0.05) and *L. sakei* treatment significantly increased the expression of *GDNF*. The expression of GDNF in both the ST and the synbiotic group was increased but did not reach a significant difference compared with the constipation group (Figure 2E). It has been reported that colonic motility is negatively correlated with the production of nitric oxide (NO) and the expression of NOS, the only enzyme involved in NO synthesis. We found that the expression level of *NOS* in the normal group was significantly lower than that in the constipation group. Both ST or *L. sakei* interventions significantly decreased the expression levels of *NOS* (*P* < 0.05) while the symbiotic intervention has less effect on the expression of NOS (Figure 2F, *P* > 0.05).

### Effects of ST or *L. sakei* alone or combined on protein levels of constipation-related intestinal factors

**Figure 3:**
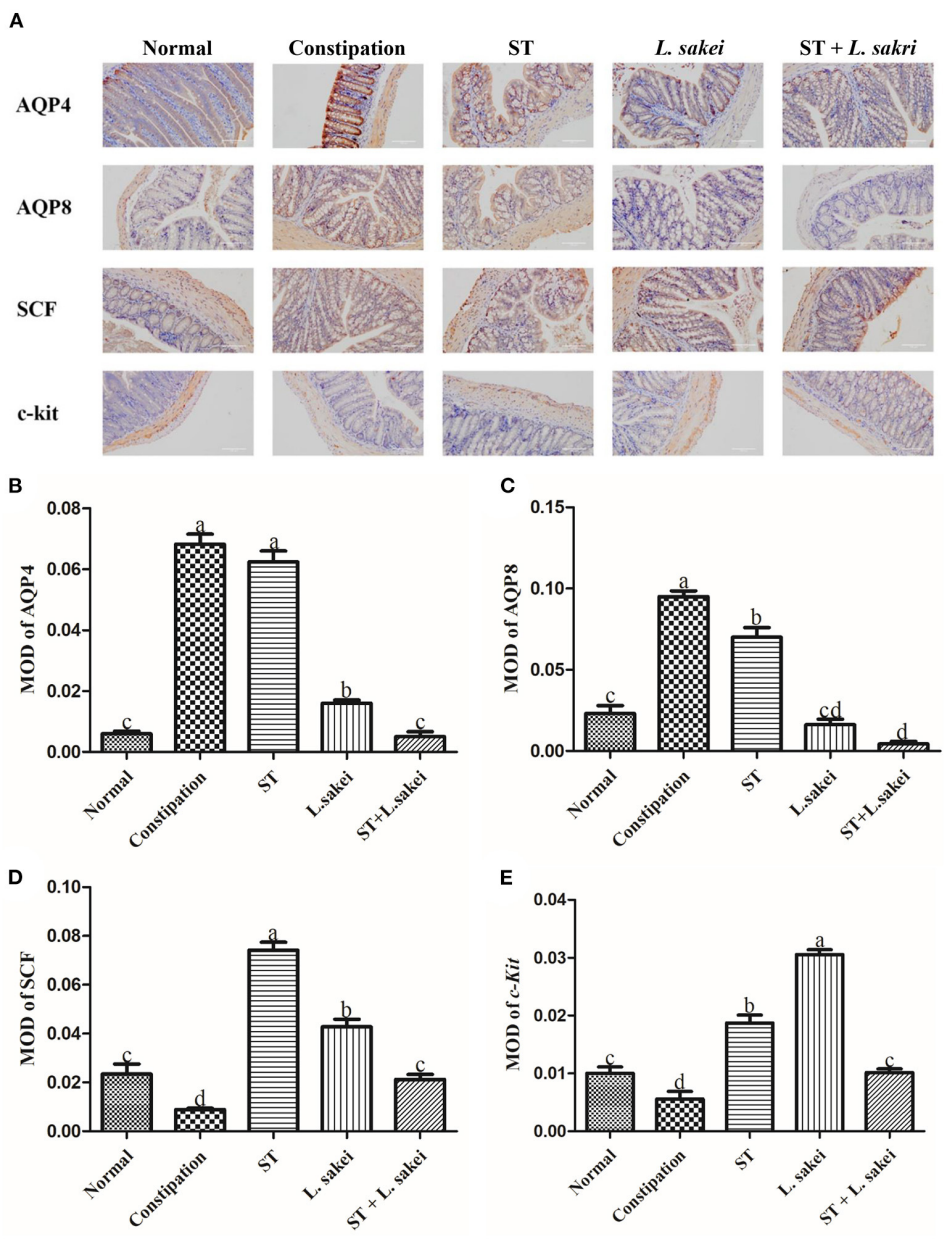
The effect of ST or *L. sakei* alone or combined on the protein levels of constipation-related intestinal factors in mice. **(A)** Protein expressions of AQP4, AQP8, c-kit, and SCF in the colons of mice were detected by immunohistochemistry and were observed under a 200×00 microscope. The mean optical density (MOD) of AQP4 **(B)**, AQP8 **(C)**, c-kit **(D)**, and SCF **(E)** was calculated and evaluated by an experienced person who was blind to the samples in terms of treatment. Data represent the mean ± SD (*n* = 6). Statistical analysis was conducted using One-way ANOVA followed by Tukey's multiple comparisons test for each group using GraphPad Prism5. Bars bearing different letters indicate a significant difference, *P* < 0.05.

Immunohistochemical results of protein levels of AQP4, AQP8, c-Kit, and SCF in colon tissue were shown in Figure 3A, which were observed under a 200 × microscope and the average optical density value was calculated. The result showed that the protein levels of AQP4 and AQP8 in the colon of constipation mice were significantly higher than those in the normal group. Compared with constipation mice, the mice in the three intervention groups all had significantly lower levels of AQP4 and AQP8 in colons except for the effect of prebiotic ST treatment on AQP4 level (Figures 3B,C, *P* < 0.05). The protein expressions of c-Kit and SCF in the colon of constipation mice were significantly lower than those in the normal group, and the down-regulation of c-Kit and SCF by constipation was increased in all three intervention groups (Figures 3D,E, *P* < 0.05). These results indicate that intervention of ST or *L. sakei* alone or combined could affect the protein level of aquaporins and the number of ICCs to relieve constipation.

### Effects of ST or *L. sakei* alone or combined on neurotransmitter and hormone levels in the serum of constipation mice

**Figure 4:**
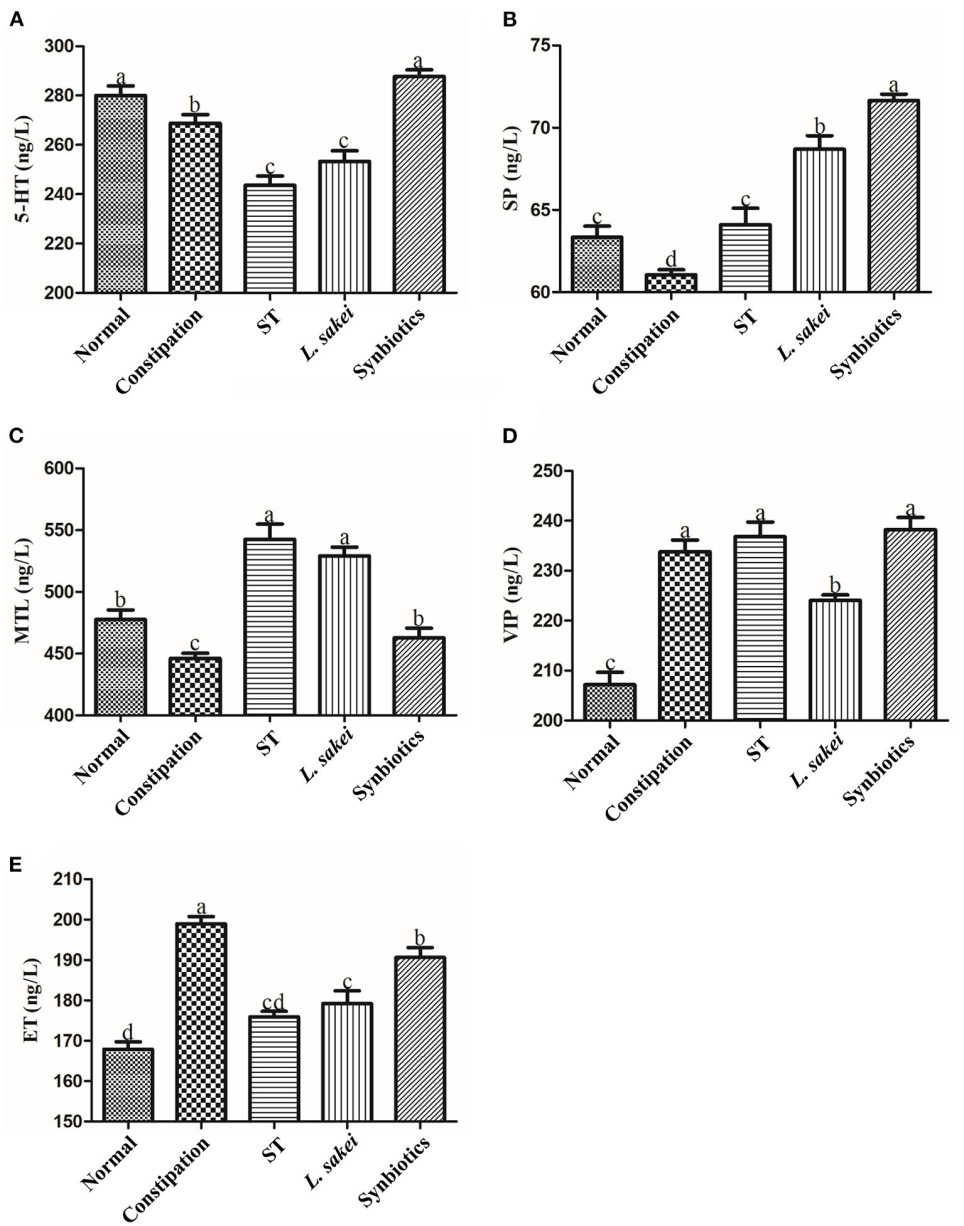
Effects of ST or *L. sakei* alone or combined on intestinal hormone levels in mice. **(A)** 5-HT level; **(B)** SP level; **(C)** MTL level; **(D)** VIP level; **(E)** ET level. 5-HT, 5-hydroxytryptamine; SP, substance P; MTL, motilin; VIP, vasoactive intestinal peptide; ET, endothelin. Statistical analysis was conducted using One-way ANOVA followed by Tukey's multiple comparisons test for each group. Bars bearing different letters indicate a significant difference, *P* < 0.05.

By measuring the excitatory neurotransmitters serotonin (5-HT), substance P (SP), and the gastrointestinal hormone motilin (MTL), and the inhibitory neurotransmitters vasoactive intestinal peptide (VIP) and endothelin (ET), we explored whether treatment with ST or *L. sakei* alone or combined can restore intestinal function by affecting the content of intestinal neurotransmitters. The results showed that the levels of excitatory neurotransmitters and hormones including 5-HT (271.32 ± 10.16 ng/L); SP (61.05 ± 0.76 ng/L), and MTL (449.88 ± 11.25 ng/L) in the serum of constipation mice were significantly lower than those in the normal group (5-HT 280.14 ± 8.74 ng/L; SP 63.35 ± 1.65 ng/L; MTL 473 ± 17.1 ng/L, *p* < 0.05). Among the three intervention groups, only the symbiotic intervention significantly increased the content of 5-HT (287.34 ± 10.16 vs. 271.32 ± 10.16 ng/L). In addition, three interventions all increased the production of SP (ST 64.1 ± 2.46 ng/L, *L. sakei* 68.69 ± 2.02 ng/L, and Symbiotic 71 ± 0.94 ng/L vs. PBS 61.05 ± 0.76 ng/L) and MTL (ST 548.52 ± 28.26 ng/L, *L. sakei* 523.24 ± 20.25 ng/L, and Symbiotic 457.69 ± 20.05 ng/L vs. constipation 449.88 ± 11.25 ng/L; Figures 4A–C). We noticed that the synbiotic intervention had the strongest up-regulation effect on 5-HT and SP. For inhibitory neurotransmitters, the levels of VIP (233.81 ± 5.26 ng/L) and ET (198.98 ± 4.36 ng/L) in the serum of constipation mice were increased compared with normal mice (VIP 207.16 ± 6.12 ng/L, ET 167.87 ± 4.57 ng/L). Among the three intervention groups, only *L. sakei* intervention down-regulated the expression of VIP (224.08 ± 2.43 vs. 233.81 ± 5.26 ng/L; Figures 4D,E). Administration of ST or *L. sakei* affected all the above-mentioned neurotransmitters except VIP. All three interventions restored and decreased the content of ET. Our data showed that the administration of ST or *L. sakei* alone or combined can differently affect the production of neurotransmitters and hormones.

### Effects of ST or *L. sakei* alone or combined on short-chain fatty acids (SCFAs)

**Figure 5:**
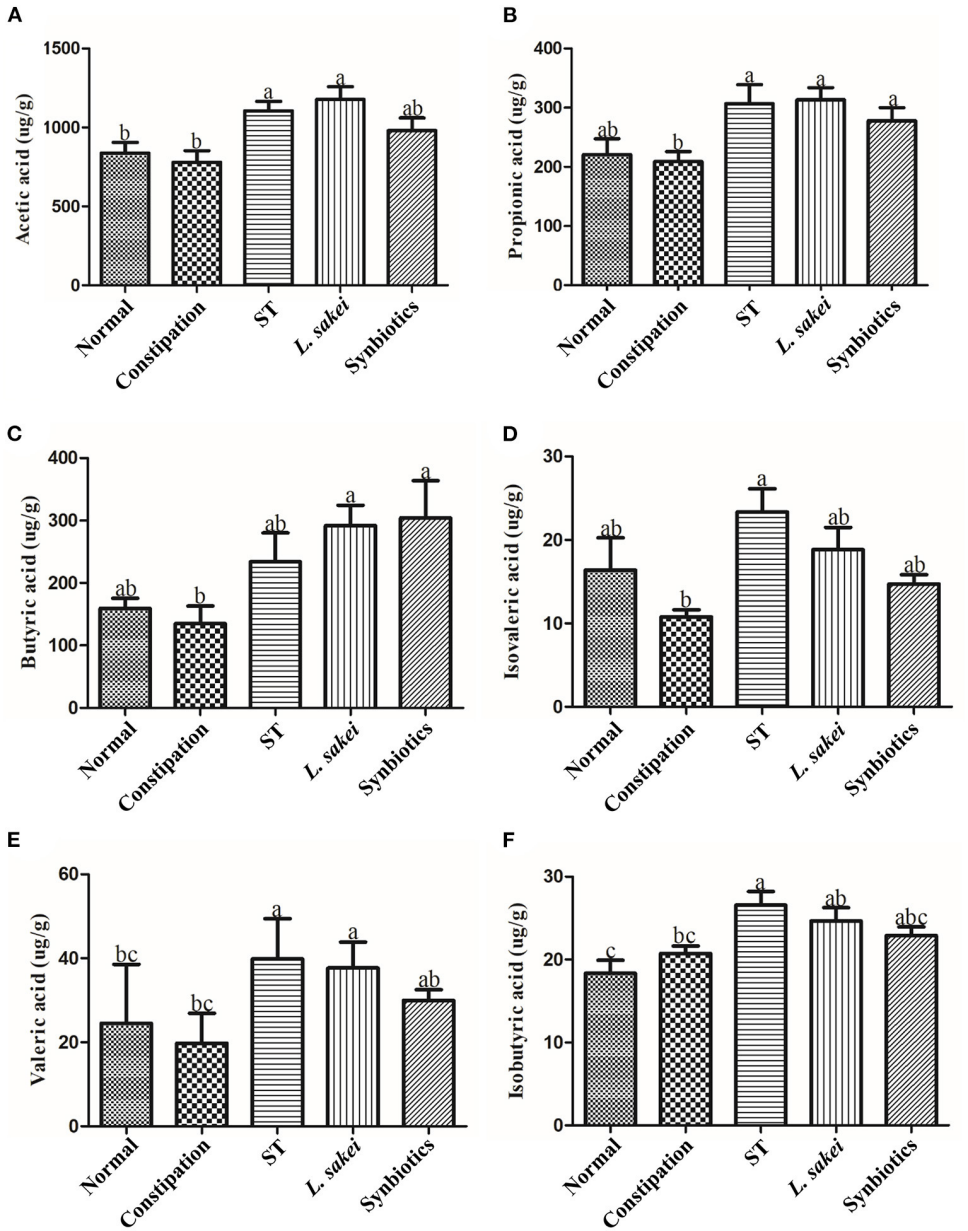
The effect of ST or *L. sakei* alone or combined on short-chain fatty acid levels of constipation-related intestinal factors in mice. **(A)** Acetic acid; **(B)** Propionic acid; **(C)** Butyric acid; **(D)** Isovaleric acid; **(E)** Isobutyric acid; **(F)** Valeric acid. Data represent the mean ± SD (*n* = 6). Statistical analysis was conducted using one-way ANOVA followed by Tukey's multiple comparisons test for each group. Bars bearing different letters indicate a significant difference, *P* < 0.05.

The abundance of SCFAs in the cecal content of all mice was measured in this study. The results showed that there was no significant difference in the abundance of SCFAs between the normal group and the constipation group. The mice supplemented with ST had a significantly higher amount of acetic acid (1105.87 ± 147.84 μg/g), propionic acid (306.86 ± 78.3042 μg/g), isovaleric acid (23.33 ± 6.91 μg/g), isobutyric acid (26.56 ± 4.03 μg/g), and valeric acid (39.81 ± 8.79 μg/g) while the mice supplemented with *L. sakei* had a significantly higher amount of propionic acid (313.53 ± 49 μg/g), acetic acid (1177.42 ± 198.4 μg/g), butyric acid (291.86 ± 80.05 μg/g), and valeric acid (37.69 ± 5.65 μg/g) compared with constipation mice treated with PBS (acetic acid 779.83 ± 178.15 μg/g, propionic acid 208.90 ± 41.51 μg/g, isovaleric acid 10.79 ± 1.87 μg/g, isobutyric acid 20.73 ± 2.22 μg/g, butyric acid 134.76 ± 69.49 μg/g, valeric acid 19.78 ± 6.50 μg/g). Administration of synbiotics only increased the amount of propionic acid (277.87 ± 54.47 μg/g) and butyric acid (304.33 ± 145.85 μg/g; Figures 5A–F). These results showed that both ST or *L. sakei* alone or combined could relieve constipation by increasing the abundance of SCFAs.

### Effects of ST or *L. sakei* alone or combined on the diversity of gut microbes

**Figure 6:**
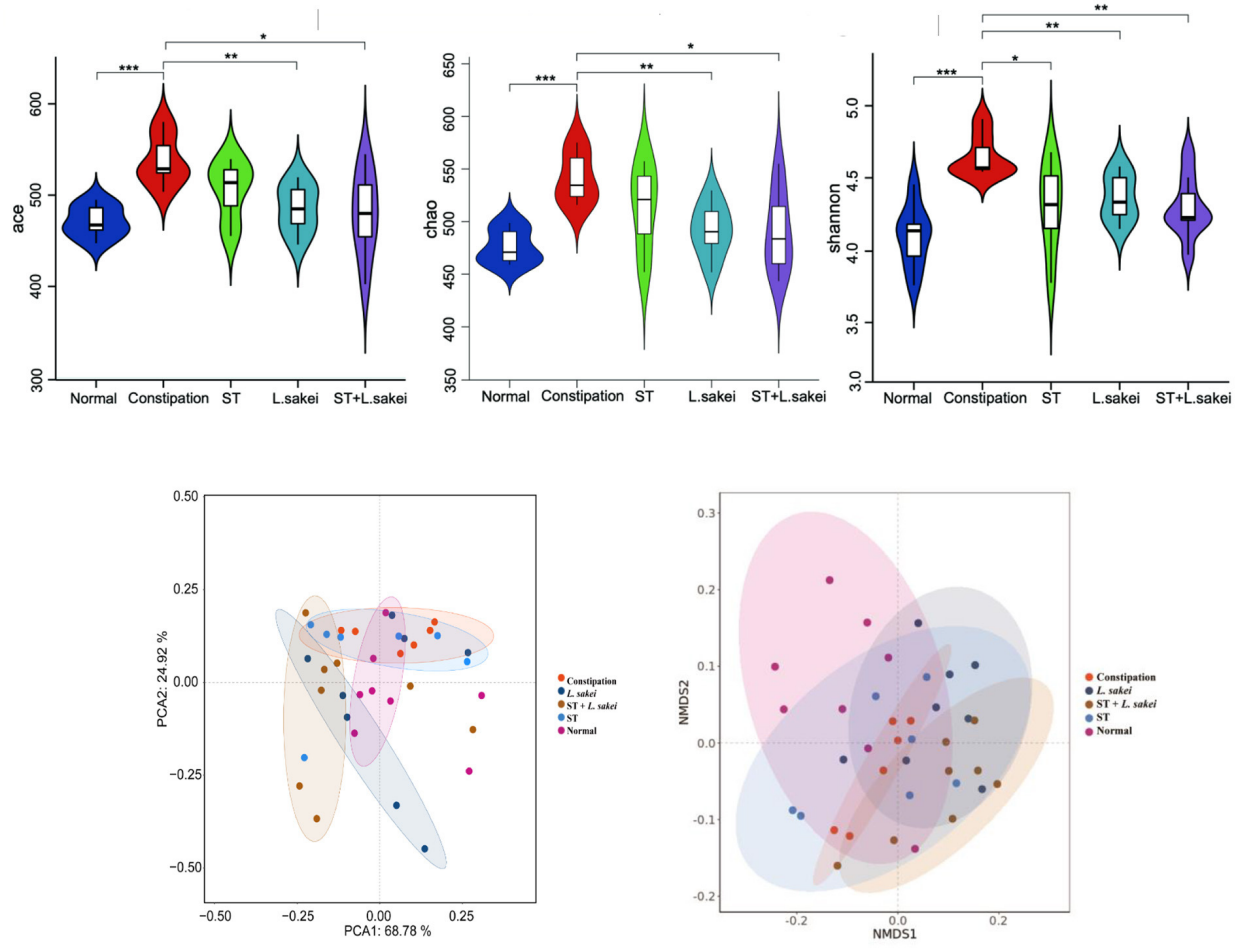
The effect of ST or *L. sakei* alone or combined on the alpha diversity analysis and beta diversity analysis in mice microbiota. **(A)** Ace index; **(B)** Chao index; **(C)** Shannon index; **(D)** PCA score plot at the phylum level; **(E)** NMDS plot at the genus level. Data represent the mean ± SD (*n* = 6–8). Wilcoxon signed-rank test of non-parameter statistics was used for constipation group vs. normal group, ***p < 0.001. Wilcoxon signed-rank test of non-parameter statistics was used for ST, *L. sakei*, and ST + *L. sakei* groups, vs. constipation group *p < 0.05, **p < 0.01.

The effects of ST and *L. sakei* on the diversity of gut microbes were analyzed. As shown in Figures 6A–C, the α-diversity of gut microbes in the constipation group was significantly greater than that in the normal and three intervention groups. It indicates that intervention with ST or *L. sakei* could reverse the changes in the diversity and species richness in mice caused by constipation. The beta diversity of gut microbes was analyzed at the phyla (Figure 6D) and genus level (Figure 6E). The gut microbes of the normal group and the constipation group were significantly different at the phylum level. Among the three intervention groups, the symbiotic group was the most different and the prebiotic group was the least different from the constipation group (Figure 6D). At the genus level, the beta diversity of gut microbes in the normal group, the constipation group, the prebiotic group, and the probiotic group was close to each other while the symbiotic group was different from the other four groups (Figure 6E), which indicates that the combination of ST and *L. sakei* could significantly change the diversity of gut microbes in constipated mice.

### Effects of ST or *L. sakei* alone or combined on the structure of intestinal microbiota in mice

**Figure 7:**
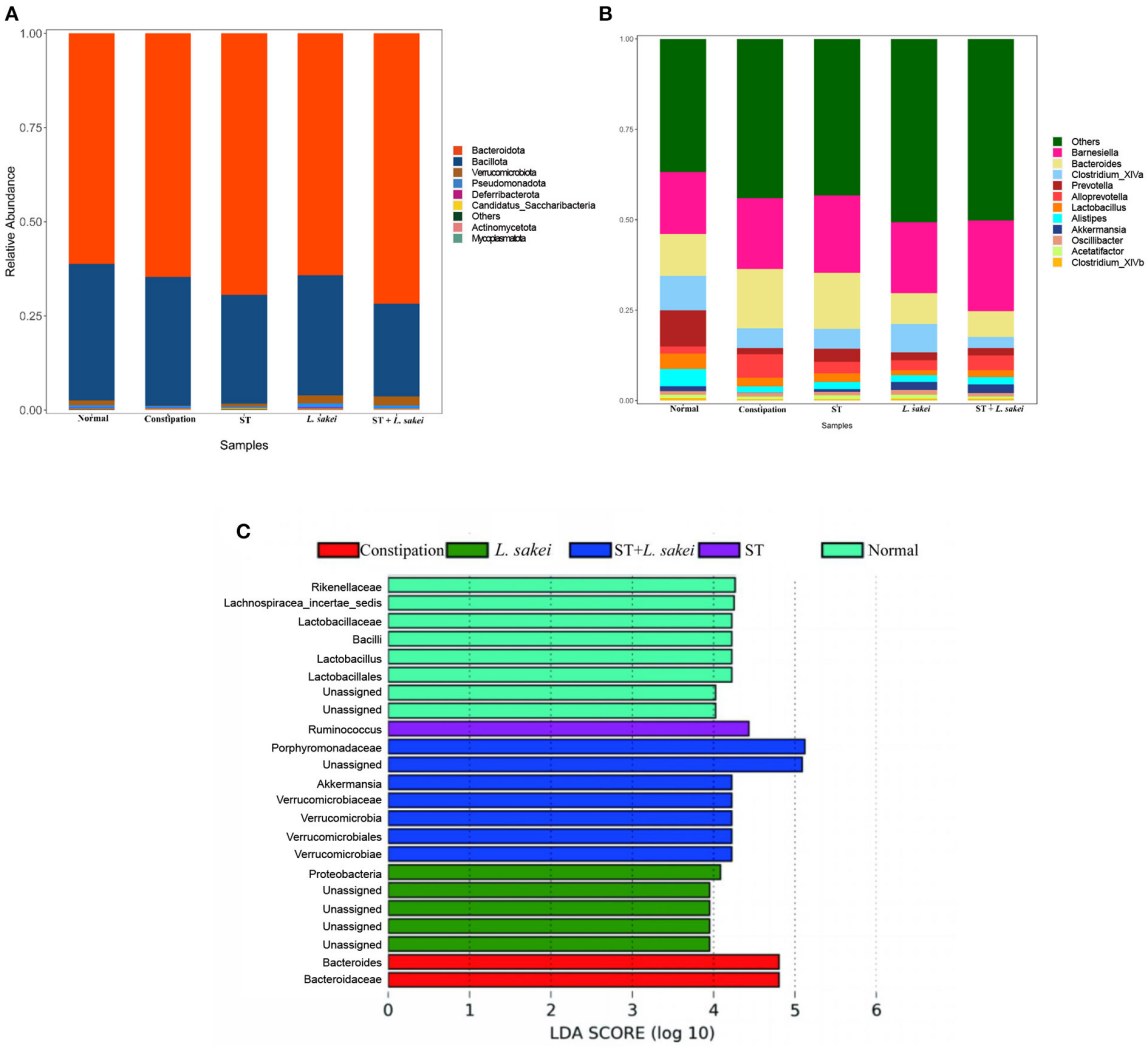
The effect of ST or *L. sakei* alone or combined on the phylum- and genus-level structures of the gut microbiota. **(A)** Relative abundance bar plot at the phylum level; **(B)** Relative abundance at the genus level; **(C)** LEfSe analysis of gut microbiota. Data represent the mean ± SD (*n* = 6–8). Wilcoxon signed-rank test of non-parameter statistics was used for constipation group vs. normal group, ***p < 0.001. Wilcoxon signed-rank test of non-parameter statistics was used for ST, *L. sakei*, and ST + *L. sakei* groups, vs. constipation group *p < 0.05.

At the phylum level, the microbiota composition and relative abundance of each group are shown in Figure 7A. The relative abundance of Verrucomicrobiota in the constipation group was significantly lower than the normal and three intervention groups. The relative abundance of Verrucomicrobiota in both *L. sakei* and ST + *L. sakei* mice was even higher than that of the ST and normal mice, indicating that intervention with *L. sakei* can significantly increase the relative abundance of Verrucomicrobiota and reverse the reduction by constipation. At the genus level, compared with the normal group, the relative abundance of *Bacteroides* and *Alloprevotella* had a trend of increasing in the constipation group, and the relative abundance of *Clostridium_XlVa, Prevotella, Alistipes*, and *Akkermansia* tended to decrease. The intervention of ST tended to partially restore the relative abundance of *Prevotella, Alloprevotella, Alistipes*, and *Akkermansia, L. sakei* tended to partly restore the relative abundance of *Bacteroides, Clostridium_XlVa, Alistipes*, and *Akkermansia*, and ST + *L. sakei* tended to partially restore the relative abundances of *Bacteroides, Alistipes*, and *Akkermansia* (Figure 7B).

To determine the difference in the abundance of species among populations, we performed the LEfSe analysis (Figure 7C). We found that the characteristic gut microbes of the normal group were Rikenellaceae, Lachnospiracea_incertae_sedis, Lactobacillaceae, *Bacilli, Lactobacillus*, and Lactobacillales. The characteristic gut microbes of the constipation group were *Bacteroides* and Bacteroidaceae. The characteristic gut microbes of the prebiotic group were *Ruminococcus*. The characteristic gut microbes of the probiotic group were *Proteobacteria* and the characteristic gut microbes of the synbiotic group were Porphyromonadaceae, *Akkermansia*, Verrucomicrobiales, Verrucomicrobiota, Verrucomicrobiaceae, and, Verrucomicrobiae. Together, these results suggest that the combination of ST and *L. sakei* probably relieves constipation by altering the abundance of beneficial bacteria and harmful bacteria.

## Discussion

Alteration in intestinal peristaltic contractions can result in prolonged intestinal transit time, prolonged bacterial fermentation time, and prolonged water absorption time, which is associated with reduced fecal water content and decreased defecation. The defecation process is associated with multiple factors including neurotransmitters, hormones, gut microbes, interstitial cells of Cajal, and intestinal motility factors (34). Abnormalities in any of these factors can lead to constipation. According to the previous studies using constipation models, we set the initial observation indicators such as the first defecation time, stool wet weight, stool quantity, stool water content, and small intestine propulsion rate in this study (35–38). Studies have shown that the beneficial effect of ST on intestinal diseases is mainly reflected in two aspects, i.e., broad effects, such as on bowel parameters and fecal water content; and specific effects, such as SCFAs and intestinal effects of neurotransmitters (30). Our results showed that three interventions with ST or *L. sakei* alone and combined all alleviated constipation, and synbiotic intervention was superior to ST or *L. sakei* alone in some defecation indicators presumably because ST not only has a laxative effect on the intestinal tract itself, but it also promotes the growth of *L. sakei* and other probiotics in the intestinal tract.

Aquaporin (AQP) channels are involved in regulating fluid homeostasis in the colon (39). The expression level of AQP in the gut is particularly important for fecal conditions (39). When the expression level of a specific AQP is too high, the gut will over-absorb water from the feces, and thus reduce the water content of the feces resulting in difficult fecal excretion and constipation (39). We detected the expression of AQP by RT-PCR and IHC and found that the expressions of AQP4 and AQP8 in the intestines of mice in the constipation group were significantly increased. Intervention of ST or *L*. *sakei* alone or combined down-regulated the expression levels of AQP4 and AQP8, and the combination of ST and *L*. *sakei* had the greatest effect on AQP expression. We, therefore, speculated that down-regulating the expression of AQP4 and AQP8 by ST or *L. sakei* may contribute to increasing the water content of feces. Downregulation of SCF and c-Kit in constipation mice could lead to a decrease in ICCs (40). ICCs are a type of intestinal pacemaker cell closely associated with the development of STC (40). In this study, ST and *L. sakei* could act on c-Kit and its ligand SCF to increase the number of ICCs, thereby promoting intestinal EMG activity and smooth muscle contraction to relieve constipation.

Studies have shown that when GDNF (glial cell-derived neurotrophic factor) is knocked out in mice, the mice lose gastrointestinal innervation, which affects intestinal motility (41). Therefore, increasing the expression of GDNF helps prevent and relieve constipation. In addition, NOS is the only enzyme involved in nitric oxide (NO)synthesis. With the increase of NOS, NO diffuses to smooth muscle cells, where it increases cyclic guanosine monophosphate (cGMP) resulting in the decrease of intracellular Ca^+^ concentration, relaxation of smooth muscle, weakness of conductance, and inhibition of gastrointestinal motility (42). Therefore, reducing the production of NO and the expression of NOS may help alleviate constipation (42). Our results showed that intervention with *L. sakei* increased the expression level of GDNF, and supplement with ST or *L. sakei* reduced the expression level of NOS, indicating that ST or *L. sakei* may relieve constipation and accelerate intestinal motility by affecting the expression of GDNF and NOS.

We measured some serum factors related to intestinal motility and found that diphenoxylate-induced constipation could reduce the expressions of 5-HT, MTL, and SP in serum and increase the expressions of ET and VIP. Ninety-five percent of 5-HT in the body is produced by enterochromaffin cells. The regulation of the intestinal system by the enteric nervous system requires the mediation of 5-HT. Large amounts of 5-HT activate primary afferent neurons in the submucosa, stimulate neurotransmitter release, and cause regular intestinal contractions (43). Therefore, 5-HT plays a major role in controlling intestinal motility. MTL is a hormone in the digestive tract and plays a role in promoting the motility of the gastrointestinal tract and the transportation of water and electrolytes in the gastrointestinal tract. Studies have found that this physiological effect is mainly through the stimulation of inter-digestive myoelectric activity, which promotes the contraction of gastric force and segmental movement of the small intestine (44). SP is a ubiquitous excitatory neurotransmitter and is usually released by intrinsic neurons or the vagus nerve in the digestive tract. SP is not only involved in the transmission of pain sensation but also binds to the receptor NK1 to regulate intestinal motility (45). On the other hand, both ET and VIP are inhibitory neurotransmitters. They are closely related to vasoconstriction. It has been reported that many elderly people with constipation also suffer from cardiovascular and cerebrovascular diseases (46). Excessive VIP levels can relax smooth muscle, and lower VIP levels can induce intestinal spasms. This study showed that the synbiotics intervention increased the levels of 5-HT, MTL, and SP, and decreased the levels of VIP and ET. This provides evidence to support the use of ST and *L. sakei* in combination as synbiotics.

SCFAs are important metabolites produced by intestinal microbiota. One study reported that treatment with loperamide led to significant reductions in the fecal levels of acetic acid, propionic acid, butyric acid, and valeric acid compared to the normal group, but there were no significant changes in fecal levels of isobutyric acid and isovaleric acid (47). Another study reported that people with constipation have significantly lower levels of fecal propionic acid and butyric acid compared with those in the normal population (48). There was no significant difference in the level of SCFAs in the cecum content between the normal and constipation mice in our study probably because 24h diphenoxylate treatment was too short to alter the metabolism of microbiota to produce SCFAs. The different profiles of SCFAs between the cecum and fecal content may be another reason for inconsistent results. Moreover, it was reported that the administration of *Lactiplantibacillus plantarum* NCU116 significantly improved the symptoms of constipation in mice and led to significant increases in acetic acid and propionic acid levels in their feces (49). But it was also reported that five strains of *L. rhamnosus* failed to recover the fecal levels of SCFAs (46). The roles of SCFAs in the alleviation of constipation remain unclear. Our results showed that both ST and *L. sakei* could slightly increase the level of SCFAs but how SCFAs play an important role in preventing constipation remain to be studied.

Gut functions are closely related to the composition of gut microbiota. At the phylum and genus levels, the relative abundance of *Bacteroidota* in the model group was increased in our study. Our result is inconsistent with one clinical study showing that *Bacteroidota* were significantly reduced in the feces of constipated children (50). The conflicting result is probably due to the different compositions of gut microbiota between mice and humans. Therefore, the relationship between *Bacteroidota* and constipation is not yet clear. By comparing the gut microbiota of African children on a predominantly vegetarian diet with those of European children on a “Western diet,” the abundance of *Prevotella* decreased significantly in the European group of children, further suggesting that *Prevotella* abundance is positively correlated with dietary fiber content and decreased abundance of *Prevotella* is associated with the onset of constipation (50, 51). Our result showed that the abundance of *Prevotella* was decreased in the constipation model group and increased after ST intervention. Therefore, we speculate that ST may relieve constipation by increasing the abundance of *Prevotella* in the intestine. Studies have found that the abundance of *Alistipes* is involved in gastrointestinal function, and can produce SCFAs and reduce intestinal inflammation (52). *Alistipes* in the three intervention groups were all increased to a certain extent. The LEfSe results showed that the characteristic gut microbiota of the ST group was *Ruminococcus*, which is known to metabolize some refractory plant components and generate SCFAs to provide energy for the host, and it was related to the relief of functional constipation (53). The relative abundance of *Akkermansia* in the constipation group decreased, and its relative abundance increased after treatment, which is consistent with previous results (54). *Akkermansia* has been widely studied to significantly improve obesity, inflammation, colon cancer, and autism (55–58), but its relationship to constipation is unclear. Some studies have found that *Akkermansia* can degrade mucin and positively regulate the thickness of the intestinal mucus layer and the integrity of the intestinal barrier (59). Therefore, we speculated that *Akkermansia* may relieve constipation by improving intestinal inflammation and repairing the intestinal mechanical barrier.

## Conclusions

Our results from the defecation experiments and intestinal propulsion experiments in mouse models suggest that oral administration of ST or *L. sakei* alone or combined are all effective to relieve constipation. Improvement of constipation may be mediated through different mechanisms of reducing intestinal inflammation, altering associated protein expression, modulating neurotransmitter release, and improving gut microbiota.

## Data availability statement

The data presented in the study are deposited in the https://www.ncbi.nlm.nih.gov/, repository, accession number PRJNA876368.

## Ethics statement

This study was approved by the Ethics Review Committee of the National Institute for Communicable Disease Control and Prevention at the Chinese Center for Disease Control and Prevention (Beijing, China).

## Author contributions

YG and ZR conceptualized the experiments and wrote the paper. YG, LS, YH, YX, and XL conducted the experiments. YG and YH analyzed the data. All authors contributed to the article and approved the submitted version.
